# Acceptability of and Willingness to Use Virtual Reality Exergames for Weight Loss Among Young Adults With Overweight or Obesity in China: Qualitative Study

**DOI:** 10.2196/66998

**Published:** 2025-05-01

**Authors:** Yanya Chen, Bingsheng Guan, Yaqi Zhang, Suen Chow Lee, Jia-yu Liu, Sicun Li, Ming Liu, Xiaoshen Zhang, Wai-kit Ming

**Affiliations:** 1 School of Nursing, Jinan University, Guangzhou, China; 2 Department of Infectious Diseases and Public Health, Jockey Club College of Veterinary Medicine and Life Sciences, City University of Hong Kong, To Yuen Building, 31 To Yuen Street, Hong Kong, China (Hong Kong), 852 34426956; 3 Department of Gastrointestinal Surgery, The First Affiliated Hospital, Jinan University, Guangzhou, China; 4 School of Public Health, University of Hong Kong, Hong Kong, China (Hong Kong); 5 Department of Cardiovascular Surgery, The First Affiliated Hospital, Jinan University, Guangzhou, China; 6 Institute of Global Governance and Innovation for a Shared Future, City University of Hong Kong, Hong Kong, China (Hong Kong)

**Keywords:** acceptability, virtual reality, exergames, overweight, obesity

## Abstract

**Background:**

Overweight and obesity seriously affect physical and psychological health worldwide. They are common public health issues in young adults who are considered a “vulnerable group” prone to adopt unhealthy lifestyles that can lead to overweight and obesity. Virtual reality exergames could help increase balance performance among patients with Parkinson disease and improve depression and pain interference among individuals with chronic neck pain. Still, limited research has been conducted on the use of virtual reality exergames among young adults with overweight and obesity, and their willingness and acceptability remain unclear.

**Objectives:**

This study aimed to assess the acceptability of and willingness to use virtual reality exergames for weight loss among young adults with overweight or obesity.

**Methods:**

This was a qualitative study. Sixteen young adults with overweight or obesity were recruited in Guangzhou, China, and divided into 4 focus groups. They were interviewed between September and October 2023 through semistructured interviews. NVivo (version 14; Lumivero) was used to transcribe, code, and thematically analyze interviews.

**Results:**

Four main themes and 8 subthemes emerged from the data. The main themes included perception of previous weight loss measures (regarded exercise and diet as the main measures for weight loss and difficulties in holding on to the weight loss methods), acceptability of virtual reality exergames (increasing opportunities for exercise, a powerful means for propelling doing exercise, positive impact on psychological well-being, and more appealing to office workers than students), willingness to use virtual reality exergames, and concerns (weight loss effect and other concerns).

**Conclusions:**

Virtual reality exergames were seen as beneficial for maintaining exercise and promoting psychological well-being among young adults with overweight or obesity, despite concerns about effectiveness, cost, and privacy. Almost all young adults indicated their willingness to try these kinds of games if given the opportunity. These findings suggested that virtual reality exergames could be a promising tool for weight management in this population.

## Introduction

The epidemic of obesity or overweight has gone beyond being a cosmetic concern to become a disease that poses a substantial threat to the well-being of individuals worldwide. Obesity raises the likelihood of developing a series of diseases, such as diabetes, hypertension, arteriosclerosis, hyperlipidemia, cardiovascular disease, and specific types of cancer [1]. Individuals with obesity also grapple with psychological disorders, including depression, lack of self-confidence, and low self-efficacy [2]. In 2025, more than 3041 million adults will be overweight (42% of the population), and 1249 million will be diagnosed with obesity (17% of the population) [3]. Especially, obesity prevalence among young adults ranged from 2.3% to 12%, and overweight was 28.8% [4]. Young adults (aged 18‐25 years) are considered a “vulnerable group” prone to adopting unhealthy lifestyles that can lead to overweight and obesity [5,6]. This age range coincides with significant life transitions such as starting college, getting married, and beginning to build a family, which have been associated with weight gain [7]. Therefore, it is imperative to implement treatment and intervention programs targeting weight loss among young adults.

Engaging in enjoyable and sustainable physical activities that consistently motivate young adults is crucial for promoting weight loss and sustaining a healthy weight [8]. Nevertheless, conventional forms of physical exercise, such as jogging, yoga, and aerobic workouts, often fail to captivate individuals with obesity, leading to inconsistent and infrequent exercise habits. Moreover, young adults often struggle to find the time and energy to exercise regularly due to the demands of work and study [9,10]. The term “exergaming” (a portmanteau of “exercise” and “games”) refers to technology-based physical activities that involve participants being physically active or exercising in order to play by using full-body movement as the primary means of interaction [11]. Despite potential drawbacks such as cybersickness, virtual reality (VR)-based exergames enhance involvement and immersion [5,12]. They also facilitate moderate-intensity physical activity, providing the convenience of independent use at home while overcoming common barriers such as distance and time constraints [13]. Evidence suggested that VR exergames could help increase balance performance among older patients with Parkinson disease [14] and improve depression and pain interference among individuals with chronic neck pain [15]. A study conducted in Taiwan, China, demonstrated that VR exergames could help reduce tension and induce calmness in patients with incomplete spinal cord injuries, thereby aiding their rehabilitation [16]. Among individuals with obesity, VR could effectively decrease the BMI levels of overweight middle-aged women and children, while also improving overall exercise performance [12,17]. It was also reported that Second Life (a social virtual world) could improve health self-efficacy for weight loss management among adults [18]. Still, limited research has been conducted on the use of VR exergames among young adults with overweight and obesity and their willingness and acceptability remain unclear. Consequently, in this study, the aim was to evaluate the willingness and acceptability to use VR exergames by young adults with overweight or obesity in weight loss settings through focus group interviews.

## Methods

### Participants and Study Design

This study adopted a qualitative approach using semistructured interviews to elucidate the viewpoints of the participants. Focus groups were selected as they could elicit open-ended responses, providing an in-depth understanding of individuals’ perspectives, attitudes, beliefs, and opinions. Moreover, a group had the potential to foster synergies that transcended individual interviews as new insights could be generated through active listening, interactive engagement with peers, and reflective dialogue in focus groups [19].

The inclusion criteria were young adults aged 18‐25 years, with a BMI of ≥24.0 kg/m^2^ (overweight) or with a BMI of ≥28.0 kg/m^2^ (obesity), did not have any prior firsthand experience with VR exergames, voluntary participation, living in Guangzhou, and have at least a high school education. Participants were recruited through posters at a university and a community located in Guangzhou, China. While sample sizes were typically not predetermined, it was common for qualitative studies to continue data collection until reaching saturation. First, we recruited 16 participants and divided them into 4 groups of 4 participants each. To ensure that the participants in a group comfortably talk as peers with other group members, the participants were divided into groups based on BMI. Two overweight groups enrolled participants with a BMI ranging from 24.0 to 27.9 kg/m^2^, while 2 groups with obesity enrolled participants with a BMI of 28 kg/m^2^. After interviewing these participants, we opted not to recruit other participants, as we determined that the data had reached saturation.

### Interview Procedure

September to October 2023 was the collection period for the data. These were the procedures followed during the interviews: (1) Preparation: The focus group interviews were held in a quiet and isolated meeting room. The room used for the study measured approximately 20 m^2^, providing sufficient space for comfortable seating of all participants. It was well lit with natural and artificial lighting to ensure optimal visibility. The main researcher, assistant, and field notetaker arrived at the meeting room 30 minutes before the participants arrived to arrange seating and prepare recording equipment. The main researcher involved in the evaluation holds a doctoral candidate in obesity fields and has more than 6 years of experience in conducting similar studies. The assistant has a master’s degree and has participated in several research projects related to this topic. The field notetaker is an experienced researcher with a bachelor’s degree and has been trained specifically for this study to ensure accurate and reliable data collection. Participants could sit comfortably in a circle and information might be transmitted through facial expressions and body posture. Each participant was seated approximately 1‐1.5 m apart to ensure personal comfort and to maintain an inclusive and interactive environment. The main researcher, assistant researcher, and field notetaker were positioned within the circle but slightly offset to one side, allowing them to engage with participants without dominating the discussion. Before data collection, upon students’ arrival, the researcher distributed sheets containing information about this study. Following this, the researchers described the study’s purpose, methodology, and data-handling procedures in a comprehensive oral presentation. Subsequently, each participant signed the written consent form, ensuring that consent was obtained. The general characteristics of the participants were collected, including age, gender, BMI, highest education, and work status. The BMI was determined through direct measurements. Each participant’s height and weight were measured by trained research staff using the same equipment. These measurements were taken prior to the commencement of the study. (2) Introduction: The participants were explained that all obtained information would be used solely for research purposes. Once consent for recording was obtained, we turned on the portable recorder. (3) Discussion: The main researcher introduced topics based on the prepared interview-guiding questions and cultivated an environment where participants were allowed to discuss the topic freely (Textbox 1). After explaining how participants perceived their previous weight loss methods, a 7-minute Microsoft PowerPoint presentation was used to provide them with a brief overview regarding the concept and current state of VR exergames in the field of health to make sure that they had the same basic understanding. When no new contents or statements emerged, data saturation was identified; then data collection ended. Each interview took about 45‐55 minutes. To stimulate the active participation of all participants during the interview, the main research also used nonverbal communication techniques, including nodding and smiling. (4) Based on participants’ responses, the interview contents were reviewed. Each participant was provided a chance to add additional comments. After the interview, participants could receive a reward of 50 RMB (ie, US $6.83).

Textbox 1. **Questions for focus groups.**

1. Please share your feelings about your previous weight loss methods.
2. How do you feel about using virtual reality exergames to lose weight?
3. If virtual reality exercise games were used, how would they assist your weight loss?
4. What would your reaction be if you were suggested to exercise and lose weight using virtual reality exergames?
5. Is there anything you would like to add to the interview?

### Ethical Considerations

Ethics approval was obtained through the institutional review board of Jinan University (reference number: JNUKY-2023‐0128). Participant consent was obtained before interviews were conducted. This process included explaining the purpose of the research, methodology, and the procedures involved, and disclosure of compensation (participants received 50 RMB, ie, US $6.83, as reimbursement for their time). Participants could withdraw at any time without any setbacks. Furthermore, we implemented strict confidentiality measures to protect the privacy of our participants. Participants’ information was anonymously coded during the transcription of the interviews and was securely stored and accessible only to authorized research team members.

### Data Analysis

NVivo (version 14; Lumivero) software was used to code the data. Researchers systematically reviewed the transcripts, examining each sentence to gain a deeper understanding of the data and to ensure immersion before coding. The thematic analysis method described by Braun and Clarke [20] was adopted, including 6 steps totally, that is, familiarizing yourself with data, creating original codes, developing initial themes, reviewing themes, defining and naming themes, and preparing reports. Thus, we thematically analyzed the deidentified transcripts after coding. In addition, to enhance the validity of the analysis, member checking was used. After the initial coding and theme development, participants were invited to review the findings to ensure that their perspectives were accurately represented. Their feedback helped refine the themes and ensured the credibility of the interpretations. Two researchers (BG and SL) coded 2 transcripts to develop the initial coding framework. After discussing and revising the framework, 1 author (BG) coded all remaining transcripts and created categories. To ensure consistent coding across all transcripts, these researchers met regularly to further discuss the analysis, create codes, and categorize the transcripts. Subsequently, grounded in patterns of meaning in the data, emergent themes were developed and agreed upon by consensus within the main research team. To ensure transparency and reproducibility of our methods, the findings are presented in accordance with the COREQ (COnsolidated criteria for REporting Qualitative research) [21].

## Results

### Overview

A total of 16 participants (10 males and 6 females; aged 18‐24 years), with an average BMI of 27.89 (SD 3.39), were included in this study (Table 1). For the focus group interviews, participants were divided into 4 groups, with a total recording time of 198 minutes. After an initial comprehensive review of the data, a total of 64 codes were developed and 8 subthemes were identified. These subthemes were then further refined and four main themes emerged, including (1) perception of previous weight loss measures, (2) acceptability of VR exergames, (3) willingness to use VR exercise games, and (4) concerns (Table 2).

**Table 1.** Sample characteristics.

Variable	Values	
Age (years), mean (SD), n=16	21.00 (1.91)	
Sex, n (%)		
Female	6 (38)	
Male	10 (62)	
BMI (kg/m^2^), mean (SD), n=16	27.89 (3.39)	
Highest education, n (%)		
Diploma or associate	4 (25)	
Bachelor or higher	12 (75)	
Work status, n (%)		
Employed	4 (25)	
Unemployed^a^	12 (75)	

a The work status of unemployed means the state of not having a job, including students and people who are looking for jobs.

**Table 2.** Main themes and subthemes from the focus group interviews.

Main themes	Subthemes
Perception of previous weight loss measures	Regarded exercise and diet as the main measures for weight loss.
	Difficulties in holding on to the weight loss methods.
Acceptability of virtual reality exergames	Increasing opportunities for exercise.
	A powerful means for propelling doing exercise.
	Positive impact on psychological well-being.
	More appealing to office workers than students.
Willingness to use virtual reality exergames	
Concerns	Weight loss effect.
	Other concerns.

### Perception of Previous Weight Loss Measures

#### Regarded Exercise and Diet as the Main Measures for Weight Loss

Almost all participants (15 people) agreed that exercise and diet were the primary methods for weight loss, which they had used in the past. And these were the main ways they used to lose weight. Among them, 8 participants considered exercise and diet equally vital. Four reported losing weight through exercise without dietary control, while 2 noted weight loss through diet supplemented by exercise. In addition, participants also mentioned that they had used physiotherapy, acupuncture, and other crucial methods for losing weight.

*Currently, I am controlling my weight through both diet and exercise. Previously, I have tried to lose weight through exercise or diet alone, but the result was not very significant. In an attempt to control my intake of sugar and fat, I once cooked for myself in the dormitory and ended up gaining weight. Perhaps… it’s because my cooking is delicious*.

*I don’t diet much, but I exercise a lot. I play basketball quite often, and I also go swimming, but I don’t control my eating at all*.

#### Difficulties in Holding on to the Weight Loss Methods

More than half of the participants found it challenging to stick to their previous weight loss methods. The main reasons cited by the participants for the difficulty in adhering to exercise included boredom (5 participants) and dislike of the negative experiences brought by exercise (7 participants). Negative experiences mentioned included sweating and muscle soreness. Moreover, work, study, and weather were also obstacles for participants in sticking to exercise. In terms of diet, participants indicated that lack of flavor in their meals was the issue.

*Sweating makes me very uncomfortable! To clean myself, I need to walk back home and shower. But the price of walking home is too costly—I would walk a long way and sweat more!*

*Exercising alone doesn’t seem very interesting... If I had to go by myself, I definitely wouldn’t go out (to exercise).*

### Acceptability of VR Exergames

#### Increasing Opportunities for Exercise

Participants recognized that VR exergames provided them with more choices and opportunities to exercise. For example, they could exercise at home when it was difficult to exercise outside during cold or snowy winters. Furthermore, VR exergames offer an opportunity to exercise at home for those who might feel less comfortable to do exercise in public due to introversion or concerns about their body size.

*During snowy or rainy conditions, people don’t feel like going out… Yeah, just stays in one place and wraps himself in a blanket all day…. Virtual games (virtual reality exergames) can be played at home. There are simply more opportunities to exercise. Right?*

*I enjoy staying indoors by myself. I’m like a vampire who avoids sunlight, and my lifestyle habits are quite similar to those of a vampire. This (virtual reality exergames) may give me more exercise options.*

*During snowy or rainy conditions, people don’t feel like going out…Yeah, just stays in one place and wraps himself in a blanket all day… Virtual games(virtual reality exergames) can be played at home. There are simply more opportunities to exercise. Right?*

#### A Powerful Means for Propelling Doing Exercise

Many interviewees believed that VR exergames could help them maintain their exercise routines. On one hand, the fun aspect of VR exergames might motivate them to keep exercising. On the other hand, VR exergames could be played with family and friends, providing support and supervision for weight loss through exercise.

*But the game can get people moving, and then you can lose weight while playing. That’s what appeals to me the most, and it has the potential to make me stick to the exercise*.

*If I set a goal to do some exercises every day to lose weight, I might be able to stick to it on the first day. But on the second day, I might forget about it. Once I’m in bed or taking a shower, I just don’t feel like exercising. There’s nothing concrete to remind me. However, if there’s a VR headset placed nearby, seeing it might remind me of something, like doing exercises. At the very least, it would serve as a reminder for me to do this task.*

#### Positive Impact on Psychological Well-Being

Four participants mentioned that using VR exergames to lose weight might allow them to create an ideal image in the virtual world, giving them a feeling of “rebirth” and a new experience. They also felt that friendships established in the virtual world were not based on appearance, leading to increased confidence and more opportunities to make friends, which benefited their mental health. In addition, they felt less psychological burden when exercising at home in a place of relative privacy.

*In reality, there are some people who don’t like you because of your appearance, but in virtual reality, you can be reborn once again, you can shape your favorite image... I can play sports while playing games, and I have the opportunity to meet new friends through the game network, who will not discriminate against my appearance, and I will not feel inferior in the virtual world.*

*Exercising at home is causal and makes me feel relaxed. I dislike heat. But when I exercise at home using virtual reality exergames, I can basically only wear my underwear, without worrying about the eyes of others*.

#### More Appealing to Office Workers Than Students

Two participants noted that VR exergames were suitable for office workers to lose weight but might be less suitable for students. Office workers, who were often busy and tired, might find it challenging to engage in outdoor sports. Interesting VR exergames could help them reduce pressure and lose weight.

*We students have plenty of time, and there is a playground open 24 hours a day on our campus. So I prefer outdoor sports.*

*I usually get home very late and don’t have the energy to go out (for outdoor activities). If I have the opportunity to exercise in my room, for example, through virtual reality (virtual reality exergames), I would still choose to use it for the sake of my health*.

### Willingness to Use VR Exercise Games

Almost all participants indicated their willingness to try using VR exergames for weight loss if given the opportunity. Six participants preferred to use VR for weight loss exercises at home rather than outdoors. Six participants considered VR exergames as an alternative to outdoor exercise. Among these 12 people, females prefer choosing dance-related sports in VR exergames, while males tend to prefer choosing aerobic and muscle strength exercises.

*I would rather exercise at home through virtual reality exergames*.

When I exercise outdoors, I can breathe the fresh air. Outdoor exercise is my first choice….

### Concerns

#### Weight Loss Effect

Several participants expressed concerns that the intensity of VR exergames might not be sufficient, leading to unsatisfactory weight loss results.

*Does this game really work? My friend played a Nintendo(Nintendo Switch®) game, and he told me that when he was playing badminton, he did not need to swing his arm; he just needed to move his wrist with the handle, and the system would tell you that you had hit the ball. But in reality, it required us to swing our arms fully!*

*I go to the basketball court to play basketball in order to lose weight. Every time I end up sweating all over. I feel that virtual reality exergames can’t achieve that level of physical activity.*

#### Others Concerns

Some participants cited concerns beyond the effect of weight loss. Among them, 2 participants mentioned the high cost of devices, which might not be affordable for young people, including students. One participant expressed concern about the potential leakage of personal information when using VR exercise games. Another participant worried about becoming too addicted to VR exergames.

*I once thought about buying the game suite: “Ring Fit Adventure for the Switch.” But on second thought, I didn’t buy the suite because it was too expensive.*

*Addiction, yes, many computer games allow you to just sit there without moving. I just manipulate a joystick, and sometimes I end up playing the whole day. This exercise game is still a game, and it’s based on virtual reality. Could it be even more addictive?*

## Discussion

### Principal Findings

The findings from this study indicate that most young adults with overweight or obesity reported difficulty maintaining their previously used weight control methods, such as exercise and diet. This finding is consistent with the study by Rana et al [22], which reported that nearly 91.7% of participants had attempted weight loss, yet almost 28% had rarely succeeded in maintaining their efforts. As demonstrated by Dabas et al [23], extreme temperatures and prolonged rainfall have the capacity to impact individual’s travel, thereby significantly reducing the frequency of outdoor exercise and hindering the process of weight loss. As a result, some overweight people find it difficult to lose weight conventionally. In contrast to traditional exercise methods, VR games offer distinct advantages. They do not require a specific venue, and their flexible scheduling allows for easy adaptation to unexpected interruptions, such as inclement weather. Engaging in VR exergames can ensure the frequency and continuity of workouts, which helps the population with obesity adhere to their weight loss efforts. Furthermore, participants noted that negative experiences associated with exercise (muscle soreness, sweating, and fatigue) were also important factors that hindered their persistence in weight loss plans. Previous research has found that VR exergames were more engaging than standard exercise, as they can distract participants’ attention, reduce the fatigue brought about by exercise to a certain extent, and allow participants to be more engaged in enjoying the process of exercise, ignoring the negative experiences brought about by the exercise itself and enjoying the process of exercise [24].

Along with physical symptoms, young adults with overweight or obesity often experience mental health issues, including depression, low self-esteem, bullying, social isolation, and body dissatisfaction, while individuals with normal weight are less likely to experience body dissatisfaction [25,26]. In the results of this qualitative study, some participants stated that VR exergames might have the potential to improve their psychological status, aligning with previous research [27]. This seemed a common result with some previous studies that had indicated a similar impact on depression when VR exergames were used among overweight middle-aged women [28], patients with cardiovascular disease, and patients with stroke [29,30]. Virtual reality allows users to experience situations indirectly that are hard to experience. These screen-based audiovisual stimuli could arouse interest, with the psychologically positive effect of the interaction of pleasure on depression [12]. Furthermore, some VR products allow users to create avatars in the virtual world according to their personal preferences, including Second Life and Nintendo’s VR offering. Virtual reality exergames could assist young adults with obesity or overweight in improving their body satisfaction and self-efficacy [31]. Avatars in the virtual world were an opportunity to redress negative body image and perfect young adults’ low self-esteem, low self-confidence, and other issues [32]. However, it was not easy to identify the underlying mechanism responsible for the improvement of depressive symptoms since it might involve neurobiological and psychological interactions [12].

In this study, young adults with overweight or obesity recognized that VR exergames were effective in engaging young adults with overweight or obesity in sustained physical exercise, aligning with the results of previous research [32]. Weight control was regarded as a big challenge for people with overweight or obesity who attempted to lose weight and it needed ongoing dietary, physical activities, and psychological support. One reason why young adults with overweight or obesity have a relatively high acceptability and willingness to use VR exergames might be that they considered that playing VR exergames socially with peers could increase enjoyment and intrinsic motivation for exercise, which was identified in similar research [33-35]. The degree of enjoyment derived from an activity has been recognized as a predictor of the efficacy of an exercise program, leading to interactive technology-based exercise becoming the all-time most favored approach for promoting physical activity [36]. Most VR exergames, including Nintendo Switch and Kinect, could be used in the home environment, and it was easy to configure [37] while providing both single-player mode and multiplayer mode. Virtual reality has already been defined as a motivating and enjoyable form of treatment, allowing them to interact more with family members, other peers, and patients [36]. Researchers found that playing VR exercise games with companions could improve social support and self-esteem, thereby motivating and maintaining physical activity [8]. When people engaged in VR exergames, they might gain satisfaction in performing well and competing with other players, which was related to the impact of VR exergames on the activation of the mesolimbic dopaminergic pathways and their influence on the reward system of the brain [8,38]. Another reason for young adults’ acceptance of VR exergames might be the potential of VR exergames to break through the obstacle of traditional outdoor sports, which could be limited by time and space. Virtual reality exercise games provide the possibility of indoor exercise, creating convenient conditions for patients to insist on exercise [13]. In particular, generation Z (born between 1997 and 2012), aligning with our study’s focus on young adults, grew up in an environment of rapidly changing technologies and were highly adept at using digital technology [39], which influences their acceptability and willingness with VR exergames. Thus, VR exergames could become a potential way to increase physical exercise for people who were less likely to participate in regular aerobic exercise.

Although young adults with overweight or obesity thought that VR exergames could stimulate the intrinsic motivation to exercise and improve their psychological status, they still had some concerns, including the weight loss effect and the cost of the device. In the aspect of the weight loss effect of VR exercise games, Bosch et al [40] performed a study to evaluate the cardiorespiratory benefits of 30 minutes of boxing with 1 VR exergame (ie, Wii) compared with treadmill exercise in healthy young adults aged 23-27 years. It was concluded that VR exergames could provide sufficient aerobic activity for young adults. In addition, in line with previous findings [41,42], the acceptance of VR technology tended to be high among individuals with overweight or obesity as all participants in this study stated that they were willing to try to use VR exergames, although they did not have any experience of VR and had expressed worries about weight loss effect. This might be attributed to the playful nature of VR game exercises. Moreover, a previous study reported that only 1 out of 136 respondents with obesity had used VR or VR exergames as part of their obesity [42]. The low adoption rate of VR technology in obesity treatment is likely attributable to the high costs associated with VR hardware and software development [42]. However, as technology develops, the cost of VR may decrease, so the cost will not be one important factor influencing whether people use VR exergames in the future.

### Limitations

This study had several limitations. The first limitation was that our participants did not have any prior experience with VR exergames, and thus, our focus group interviews were related to a hypothetical VR exergames that they had not experienced, although a brief overview regarding the concept and current state of VR exergames in the field of health was provided. After participating in an actual game, their perspectives would change and be different. However, previous research results indicated [42] that patients with obesity have limited knowledge about VR technology, with only 1 out of 136 participants considering VR technology as part of obesity treatment. That means this study’s results can reflect most patients’ views. In future research, opportunities can be provided for patients to attempt weight loss through VR exergames, and their perspectives can be explored through qualitative research. Another limitation is the limited generalization due to typically small and nonrandom samples. While samples for quantitative studies are selected from a population subgroup that is statistically representative of the population in order to generalize results, qualitative researchers recruit participants with experience of the phenomenon of interest as well as “information-rich cases” for in-depth analysis. Its focus is often on depth rather than breadth, limiting the ability to apply results universally. In future research, these limitations should be addressed by using a larger sample size and mixed methods, such as surveys and focus groups. However, this is the first study to investigate the acceptability of and willingness to use VR exergames by young adults with overweight or obesity in China. Our findings suggest that VR exergames are generally acceptable and have the potential to be used as a tool in weight loss interventions for young adults with overweight or obesity in China. Clinicians could consider incorporating these games into weight loss programs as a way to enhance engagement and motivation among young adults.

### Conclusions

Compared with traditional weight loss methods, which were challenging to maintain, young adults with overweight or obesity considered VR exergames beneficial for maintaining regular exercise and promoting positive psychological well-being. Despite concerns regarding weight loss effectiveness, cost, and privacy issues, participants showed relatively high acceptance and willingness to engage with these games in weight loss settings. These results highlighted the potential of VR exergames as an engaging tool for weight management among young adults.

## Abbreviations

COREQ: COnsolidated criteria for REporting Qualitative research
VR: virtual reality
